# History of accidental hypothermia^☆^

**DOI:** 10.1016/j.resuscitation.2010.09.465

**Published:** 2011-01

**Authors:** Henry Guly

**Affiliations:** Derriford Hospital, Plymouth PL6 8DH, UK

**Keywords:** Hypothermia, History of medicine, Core temperature, Temperature measurement

## Abstract

Death from exposure to cold has been recognised for thousands of years but hypothermia as a clinical condition was not generally recognised until the mid-20th century and then only in extreme conditions such as immersion in cold water or snow. In the UK, hypothermia in less extreme conditions was not generally recognised until the 1960s. Recognition of hypothermia required the temperature to be measured and this did not become a clinical tool until the late 1800s and it was not used routinely until the early 1900s. Although John Hunter and James Curry did some physiological experiments in the 1700s, detailed physiological experiments were not done until the early 20th century and the use of therapeutic hypothermia for malignancy and in anaesthesia in the 1930s and 1940s provided more impetus for investigating the physiology of hypothermia in humans and familiarising the medical profession with measuring core temperatures.

## 1 Introduction

In the literature of the Heroic Age of Antarctic exploration (1895–1922) it is striking that there is no mention of hypothermia. The word itself was never used and the only medical reference to anyone suffering from hypothermia is a brief mention in Marshall's medical report on Shackleton's first expedition. He wrote that “on the southern journey … our temperatures were subnormal”.^1^ Wild says that when Marshall “took … our temperatures, his clinical thermo was not marked low enough to take any except mine. The other three were therefore below 94.2° which spells death at home”.^2^ This equates to 34.6 °C and presumably was an oral or axillary temperature and so was only mild hypothermia but this comment indicates that it was known that people could die if their temperature became too low.

The only medical description of hypothermia in the medical reports of the expeditions is a brief description by Gourdon (who went on the two French expeditions) of what he calls “congelation generale” which he describes as being rare.^3^

And yet hypothermia almost certainly occurred. On Scott's second expedition, Atkinson got lost in bad weather. Scott wrote: “He was badly frostbitten … and though a good deal confused, as men always are on such occasions, he was otherwise well… His tale is confused, but … the fact that he did not [hit land as he intended], but attempted to wander straight on, is clear evidence of the mental condition caused by that situation. There can be no doubt that in a blizzard a man has not only to safeguard the circulation in his limbs, but must struggle with a sluggishness of brain and an absence of reasoning power which is far more likely to undo him… It is a rambling tale to-night and a half thawed brain”.^4^ Not only does this seem to describe hypothermia but Scott recognised the behaviour of a cold person.

The aim of this paper is to describe the history of hypothermia, exploring why it was accepted that people could die of cold and yet hypothermia does not seem to have been recognised as a disease.

## 2 Types of hypothermia

Three types of accidental hypothermia are recognised. Acute hypothermia (often called immersion hypothermia) is caused by sudden exposure to cold such as immersion in cold water or a person caught in a snow avalanche. Exhaustion hypothermia is caused by exposure to cold in association with lack of food and exhaustion such that heat can no longer be generated. Chronic hypothermia comes on over days or weeks and mainly affects the elderly. Mixed forms also occur, e.g. the exhausted soldier who collapses into snow.

## 3 History of hypothermia

All three forms of hypothermia appear to have been recognised since ancient times. In 492 BC Mardonios, a Persian general, was sailing against the Greeks when he encountered bad weather, losing about 300 ships and 20,000 men. Herodotus, said that “… some were seized by these [sea monsters] and so perished, while others were dashed against the rocks; and some of them did not know how to swim and perished for that cause, others again by reason of cold”.^5^

Hippocrates is often quoted as describing hypothermia. For example: “cold causes fits, tetanus, gangrene and feverish shivering fits … Cold is bad for the bones, teeth, nerves, brain and the spinal cord…”.^6^

This is capable of several interpretations and it must be uncertain whether Hippocrates was describing hypothermia, but death from cold has been known for over 2000 years. This was particularly recognised by the military. In 218BC Hannibal lost 20,000 men crossing the Alps and cold injuries were described in many other military campaigns including, for example, the American War of Independence, The American Civil War and the Crimean War,^7,8^ though many of the early descriptions of cold injury did not differentiate between frostbite and hypothermia.

Perhaps the best-known example of the disruption of a military campaign by cold was Napoleon's retreat from Moscow in 1812. In this campaign the hypothermia was superimposed on hunger, exhaustion and disease. Many would have had frostbite. The best-known descriptions of cold injury are by Napoleon's surgeon, Larrey^9^ but perhaps the best descriptions of hypothermia are by Moricheau-Beaupré, another French doctor.^10^ He describes the fate of the soldiers: “some, pale and depressed by inanition swooned away and died, stretched on the snow. Others … were seized by shivering to which quickly succeeded languor and propensity to sleep. They were seen walking insensible and ignorant where they went: scarcely could you succeed in making them understand a few words … In a word, when no longer able to continue walking, having neither power nor will, they fell on their knees. The muscles of the trunk were the last to lose the power of contraction. Many of those unfortunates remained some time in that posture contending against death. Once fallen, it was impossible for them with their utmost efforts to rise again … Their pulse was small and imperceptible; respiration, infrequent and scarcely perceptible in some, was attended in others by complaints and groans. Sometimes the eye was open, fixed, dull, wild, and the brain was seized by quiet delirium…”.^10^ Later in his book he describes a condition that he calls “general asphyxia from cold” in similar words.^11^

The fact that the elderly are prone to cold has also been recognised for thousands of years. One of the Hippocratic aphorisms says: “Old men have little warmth … for this reason, fevers are not so acute in old people for then the body is cold”.^12^

This was also described in the Old Testament: “Now King David was old and stricken in years; and they covered him with clothes, but he gat no heat. Wherefore his servants said unto him, Let there be sought for my lord the king a young virgin: and let her stand before the king, and let her cherish him, and let her lie in thy bosom, that my lord the king may get heat. So they sought for a fair damsel … and brought her to the king. And the damsel was very fair, and cherished the king, and ministered to him: but the king knew her not”.^13^

It has long been known that the drunkard staggering home who collapsed in the snow would probably die and deaths from exposure to cold were collected in official statistics. In the USA, in 1860, exposure to cold made up 0.7% of violent deaths and in Scotland there were 58 deaths from cold in1876 (1.94% of violent deaths).^14^

It was also recognised that hypothermia could mimic death. Moricheau-Beaupré says that “General asphyxia … presents the image of perfect death; but persons found senseless and deeply benumbed have been recalled to life after twenty-four or forty-eight hours”^11^ and this is also shown by a book title: *Observations on apparent death from drowning, hanging, suffocation by noxious vapours, fainting-fits, intoxication, lightning, exposure to cold etc etc*. In it, Curry states that in “apparent death occasioned by excessive cold … animation [has been] brought about after having been suspended for several hours…”.^15^

Hypothermia could not be diagnosed before temperature measurement was a clinical tool. An example was a man of 45, described in 1859, who went out in a snow storm and when he returned, was shivering, cold and confused, with a poor attention span and a weak pulse of 60 per minute. He had not been drinking and a diagnosis was made of delirium tremens caused by cold and wet, from which he recovered.^16^

## 4 Measurement of temperature

Although the first thermometer for clinical use was made in 1612, it was not a practical tool until Fahrenheit invented the mercury thermometer in 1714. There were some pioneers who measured temperature in diseases^17^ but there are descriptions of thermometers being a foot long and it requiring 20 min to take a temperature. Also there were problems with ensuring accuracy.^18^ Two events in the second half of the 19th century made the thermometer a useful clinical tool.

The first was the invention in 1866 of a small thermometer that only required 5 min to obtain a temperature.^17^ The second was the publication in 1868 of Wunderlich's *Das Verhalten der Eigenwanne in Krankheiten* (translated into English in 1871) which presented data on nearly 25,000 patients and analysed temperature variations in 32 diseases.^19^ This defined the role of the thermometer as a diagnostic aid but the interest was mostly in fever and the way it varied. However, Wunderlich recognised the problems of a low temperature: “temperatures much below 36 °C [96.8 °F] are ‘collapse’ temperatures. Below 33.5 °C [92.3 °F], deep, fatal algide collapse; 33.5–35 °C [95 °F], algide collapse with great danger, still with possibility of recovery; 35–36 °C, moderate collapse, in itself without danger”.^19^ He was primarily talking of low temperatures in disease though he recognised that “extreme degrees of ‘external cold’ are the most certain means of abstracting warmth from the body; it may go so far as to render death inevitable”.^20^

Measuring axillary temperatures was the usual method until the late 1890s when antiseptics were better, allowing oral temperatures to be taken.^17^

The word hypothermia seems to have originated in the late 19th century. The first use in the British Medical Journal seems to have been in 1880 describing hypothermia in typhoid^21^ and most of the early references to hypothermia relate diseases such as typhoid,^22^ cholera,^23^ pneumonia,^24^ diphtheria^25^ and spinal cord injury.^26^

Low body temperature due to exposure to cold was described very early in the history of clinical thermometry. In 1875 Reincke described 17 men exposed to extreme cold while intoxicated. Of five with temperatures below 30 °C [86 °F], only two survived, one with a temperature of 24 °C [75.2 °F].^27^ The first review of hypothermia I have found was in 1900 but it does not differentiate between medical causes and accidental hypothermia.^28^

Accidental hypothermia is not mentioned in Osler's textbook of medicine of 1907,^29^ perhaps the standard medical textbook of the time.

The history of hypothermia is complicated not only by the different names it has been called but also because “hypothermia” has had different meanings, being used to describe “a persistent subjective coldness of the hands and feet” and “a local feeling of chilliness”^30^ and cold intolerance.^31^

## 5 Treatment

Moricheau-Beaupré recommended: “we must not … transport the body into a heated place, or immediately apply to it warm substances; too strong re-action might exhaust the remaining vitality”^11^ and “Not withstanding the greatest probability of ill-success, we must always afford the assistance described … We begin by placing the body asphyxiated in a place where there is no current of air, and the temperature is a little above that of the atmosphere; it is quickly stripped of clothes and laid on a mattress or horse-bed. Frictions with some exciting tincture are made on the precordial region and navel, and warm clothes are subsequently applied. Afterwards we proceed to the use of snow, iced water, and water successively less cold, in the same order and degree as in local asphyxia [i.e., frostbite]. This first operation should last almost a quarter of an hour; in the second place, water a little warmed and afterwards lukewarm, and hot water.

When respiration and circulation are sensibly restored, and the muscles lose something of their stiffness, and a little heat is manifested, then the body is quickly wiped with dry linen; dry frictions are made with flannel, and the patient is placed in bed, wrapped up in a woollen blanket.” Later other stimulants might be used, e.g. tickling the nostrils with a feather, frictions on the palm or sole with strong vinegar and in severe cases “some success might perhaps be obtained from the employment of the voltaic pile” (i.e., electrical stimulation with a battery).

As soon as they can swallow they could be given an infusion of tea or elderflowers “with the addition of some drops of ammonia, or a little brandy – or cinnamon wine, sweetened – by spoonfuls”.^32^

Curry's^15^ recommended treatment was very similar though earlier he had said that “the best mode of counteracting the cold, was to apply a bladder, with hot water, to the pit of the stomach”.^33^The problems of rapid re-warming of hypothermic casualties were obviously recognised but not addressed appropriately by physicians at that time. Alcohol was thought to be helpful because it caused the casualty to feel warm but in 1805 Curry said that “spirituous liquors … are, I believe uniformly hurtful, when taken under severe and continued cold”.^34^

Moricheau-Beaupré recognised that soldiers who got drunk on the retreat from Moscow, died.^35^ He thought this was because they fell asleep in the snow but by the late 19th century it was realised that the peripheral vasodilation caused heat loss and worsened the hypothermia.^36,37^ Also as vasodilatation causes a feeling of warmth, people take less care in protecting themselves against the cold. Despite this, brandy was still regarded as a treatment for cold and as late as 1915 rum was being issued to soldiers in wet and cold weather.^38^

## 6 Physiological experiments and modern appreciation of hypothermia

Physiologists started to investigate the effects of cooling animals in the 18th century. The best known was John Hunter who starting experiments in 1766 to try to discover whether animals exposed to extreme cold could recover when their temperature was raised again.^39^

The first experiments on humans also started at the end of the 18th century. Curry developed an interest in the subject after a ship floundered at the mouth of the river Mersey in 1790 and it took 23 h for the 14-man crew to be rescued, by which time three were dead. He did a series of experiments on individuals who were immersed in cold water and then variously exposed to cold still air, cold wind and placed in a warm bath.^33^ In his first experiment a man had a resting sublingual temperature 98 °F [36.7 °C]. One and a half minutes after being immersed in a tank of salt water at 44 °F [6.7 °C], his temperature had fallen to 87 °F [30.6 °C]. It then rose to 93½ °F [34.2 °C] after 12 min. When he came out and was dried, his temperature fell to 87 °F [30.6 °C]. These were not, of course, core temperatures. Curry also noted a slowing of the pulse and respiration.

Detailed experimentation in animals and men really started in the first half of the 20th century,^40–44^ but very little clinical literature was produced. An exception was a paper by Britton from Canada which described the physiology of hypothermia in animals and man and put it into a clinical context. He concluded that humans were potentially able to withstand lower temperatures than previously recognised.^45^

In the late 1930s and 1940s hypothermia developed a major clinical importance which led to more research. The first reason was the use of therapeutic hypothermia. Cold had been used for the treatment of fever and other conditions^46^ but hypothermia was used in the late 1930s for the treatment of malignancy,^47,48^ intractable pain, morphine addiction, leukaemia, and schizophrenia.^49,50^

Although Herodotus had recognised death from cold in 450 BC, immersion deaths were usually described as drowning. Thus all who died in the Titanic disaster were recorded as having drowned although most, undoubtedly, died of hypothermia.^51^ In the Second World War, many crew of torpedoed ships and of aircraft shot down over the sea were immersed in cold water and died. Many were wearing life-jackets and it was recognised that they died of hypothermia. This was the stimulus for more investigation of hypothermia and led to the Nazi hypothermia experiments in concentration camps^52^ and to investigations in the USA^53^ and UK.

In the late 1940s and 1950s, a bigger incentive for researching the physiology of hypothermia and rewarming was the development of cardiac and vascular surgery. Hypothermia reduces oxygen consumption and so allowed the circulation to be cut off during a surgical procedure without causing hypoxic damage.^54–56^ More recent use of hypothermia has been to aid recovery after cardiac arrest^57^ and neonatal hypoxic encephalopathy.^58^

Although accidental hypothermia in extreme conditions was well described by the middle of the 20th century, it was not fully appreciated that it also occurred in less extreme conditions such as those experienced by hill walkers in a temperate climate. Individual cases are not particularly common and the exhaustion hypothermia was poorly recognised in civilians, at least in the UK, until a report in 1966 described 23 incidents which produced 25 deaths and 23 survivors (five of whom had been unconscious).^59^

Following a number of case reports in the early 1960s, particularly during a very cold winter in the UK in 1963, chronic hypothermia in the elderly became better recognised. This occurs gradually over days or weeks to people who were indoors with poor heating. Occasionally there are associated medical problems and medications predisposing to hypothermia. A report for the Ministry of Health led to this being better defined and recognised.^60^

## 7 Conclusion

Death from cold has been recognised for hundreds of years but the clinical syndrome of hypothermia could not be defined until temperature measurement was simple and normal temperatures defined in the late 19th century. Even then, temperatures were not measured routinely. There was a circular problem that hypothermia was not diagnosed because temperatures were not measured routinely and they were not measured routinely because the condition had not been recognised.

In addition, the diagnosis of hypothermia requires measurement of the core temperature in, for example, the rectum or oesophagus or tympanic thermometry. This requires a low-reading thermometer (for rectal measurement) or electrical methods and the technology and familiarity in the use of these needed to wait for therapeutic hypothermia in the 1940s and 1950s. The measurement of core temperature is somewhat invasive and not a routine but is done on those suspected to be at risk of hypothermia. Hypothermia was known to be associated with extreme conditions but until hypothermia in less extreme conditions was defined, there was no reason to suspect it.

## Conflict of interest statement

There are no conflicts of interest.
