# Supplementary material for: History of accidental hypothermia
Source: Resuscitation. 2011 Jan;82(1):122–5. doi: 10.1016/j.resuscitation.2010.09.465 (PMC3060344; doi:10.1016/j.resuscitation.2010.09.465)
Supplement: Supplementary file 1 [file mmc1.doc]

4494 Accid Hipoth

Resumen

La muerte por exposición accidental a frío ha sido reconocida por miles de años pero la hipotermia como condición clínica no fue reconocida hasta mediados del siglo 20 y entonces solo en condiciones extremas tales como inmersión en aguas muy frías o nieve. En el Reino Unido, la hipotermia en condiciones menos extremas no fue generalmente reconocida hasta los años 1960s. El reconocimiento de la hipotermia requirió que la temperatura fuera medida y esto no se convirtió en herramienta clínica hasta tarde en los 1800s y no se usó de rutina hasta los primeros 1900s. Aun cuando John Hunter y James Curry hicieron algunos experimentos fisiológicos en los 1700s, no se hicieron experimentos fisiológicos detallados hasta el principio del siglo 20 y el uso de la hipotermia terapéutica para enfermedades y en anestesia en los 1930s y 1940s proporcionaron más ímpetus para la investigación de la fisiología de la hipotermia en humanos y familiarización de la profesión médica con medición de temperaturas centrales.

© 2011 Publicado por Elsevier Ireland Ltd.

*Palabras clave*: Hipotermia; Historia de la medicina; Temperatura central; medición de temperatura
